# Structural Basis of the Pore-Forming Toxin/Membrane Interaction

**DOI:** 10.3390/toxins13020128

**Published:** 2021-02-09

**Authors:** Yajuan Li, Yuelong Li, Hylemariam Mihiretie Mengist, Cuixiao Shi, Caiying Zhang, Bo Wang, Tingting Li, Ying Huang, Yuanhong Xu, Tengchuan Jin

**Affiliations:** 1 Department of Clinical Laboratory, the First Affiliated Hospital of Anhui Medical University, Hefei 230022, China; lyj106@126.com (Y.L.); scx5250@163.com (C.S.); wangbo@ahmu.edu.cn (B.W.); litingting_0725@126.com (T.L.); huangying_ah2007@sina.com (Y.H.); 2 Hefei National Laboratory for Physical Sciences at Microscale, Laboratory of Structural Immunology, CAS Key Laboratory of Innate Immunity and Chronic Disease, Division of Life Sciences and Medicine, School of Basic Medical Sciences, University of Science and Technology of China, Hefei 230027, China; lyl01@mail.ustc.edu.cn (Y.L.); hylemariam@mail.ustc.edu.cn (H.M.M.); caiyingz@mail.ustc.edu.cn (C.Z.)

**Keywords:** pore-forming toxin, structure, membrane interaction

## Abstract

With the rapid growth of antibiotic-resistant bacteria, it is urgent to develop alternative therapeutic strategies. Pore-forming toxins (PFTs) belong to the largest family of virulence factors of many pathogenic bacteria and constitute the most characterized classes of pore-forming proteins (PFPs). Recent studies revealed the structural basis of several PFTs, both as soluble monomers, and transmembrane oligomers. Upon interacting with host cells, the soluble monomer of bacterial PFTs assembles into transmembrane oligomeric complexes that insert into membranes and affect target cell-membrane permeability, leading to diverse cellular responses and outcomes. Herein we have reviewed the structural basis of pore formation and interaction of PFTs with the host cell membrane, which could add valuable contributions in comprehensive understanding of PFTs and searching for novel therapeutic strategies targeting PFTs and interaction with host receptors in the fight of bacterial antibiotic-resistance.

## 1. Introduction

Plasma membrane acts as a semi-permeability barrier between the cell and the extracellular environment, and its integrity is essential for cell survival and sustainability. Therefore, disruption of the plasma membrane is considered to be one of the ancient cell-killing mechanisms by which bacteria invade humans. Pore-forming proteins (PFPs) are among such molecules that can alter membrane permeability, in which pore-forming toxins (PFTs) constitute the major class [1,2,3]. Killing target cells by PFTs is a common virulence mechanism in a wide range of pathogenic bacteria. As the largest class of bacterial toxins, PFTs are mainly produced, but not exclusively by pathogenic bacteria. However, PFPs have been identified in all kingdoms, especially in eukaryotes, as part of their immune system [2].

The remarkable feature of (and PFTs) is that they initially and generally fold into a water-soluble, monomeric structure. Upon binding to specific receptors (sugars, lipid, or proteins) in the membrane, PFPs (PFTs) then oligomerize to form transmembrane pores with refined architecture, which alter membrane permeability and induce several responses in target cells. PFPs produced by eukaryotic organisms damage the bacteria membrane by inducing complement membrane attack complex (MAC) while damaging malignant cell membrane using perforin [2]. The pore produced by PFTs alters membrane permeabilization which allows small molecules such as ATP, specific ions, or large molecules proteins to pass through. For example, in gram-negative bacteria, type III secretion systems allow the passage of effector molecules by perforating membrane. The B subunits of AB toxins allow the passage of A subunit [4,5]. Finally, pore formation in the membrane leads to cellular behavior alteration and cell death. The interaction of PFTs and the membrane contribute to bacterial growth and colonization from the host immune response.

An increasing number of related pore-forming toxins from pathogens are being studied. Demonstrating their structure and implications on host-pathogen interaction helps to clearly understand the disease mechanism. Here we reviewed the structural mechanisms of pore formation and host-pathogen interaction of PFTs at an atomic level, contributing developing novel therapeutic strategies to fight infection targeting PFTs and/or host receptors interaction.

## 2. Structural Classification and Characterization of the Pore-Forming Toxins

**Figure 1:**
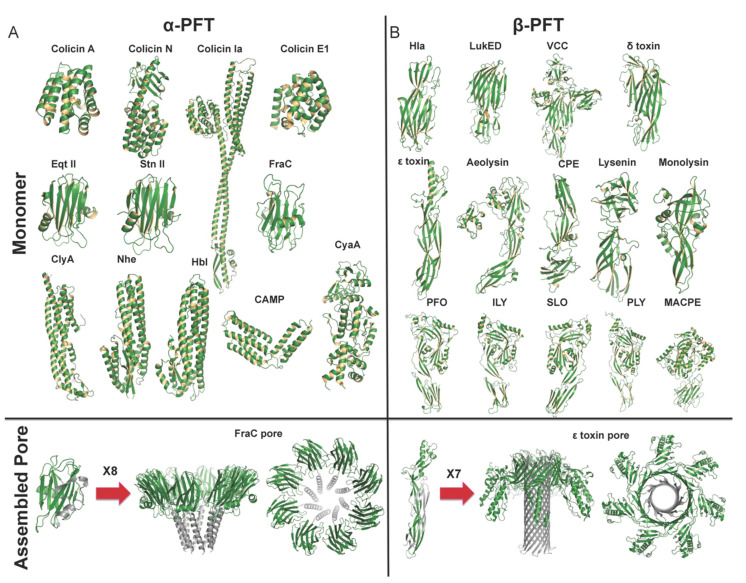
Structural characteristics of pore-formation specific for each structural family. Representative monomer and pore structure of α-PFTs and β-PFT members are illustrated. For α-PFTs, upon binding to the membrane, α-helices undergo a conformational change to insert into the membrane and form membrane pore. For β-PFTs, monomer β-PFT first assembles in a pre-stem loop, and inserts into the membrane to form a partial β-barrel, and then combines with the other protomers to form a complete β-barrel. (**A**). α-PFTs: soluble and membrane pore complex, adopted from PDBs 1COL, 1A87, 1CII, 2I88, 1IAZ, 1GWY, 4TSL, 1QOY, 4K1P, 2NRJ, 6JLC, 2COL, 4TSY. (**B**). β-PFTs: soluble and membrane pore complex, adopted from PDBs 4YHD, 4Q7G, 1XEZ, 2YGT, 1UYJ, 3C0M, 3ZIW, 3ZXD, 4MKO, 1PFO, 1S3R, 4HSC, 5AOF, 2QP2, 6RB9.

**Figure 2:**
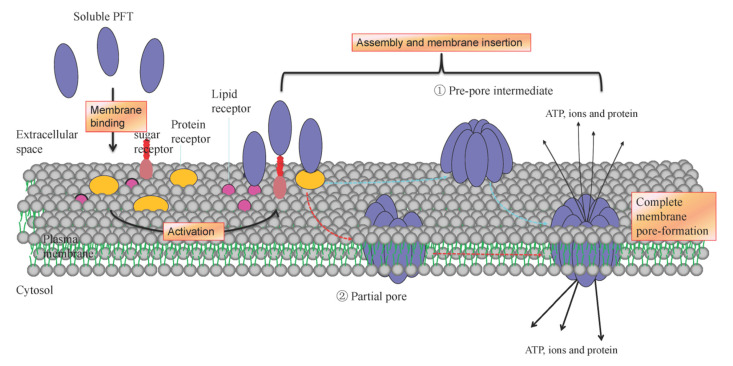
General mechanism of membrane pore formation by the bacterial PFTs. The diagram schematic presents the pore formation pathways of pore-forming toxins (PFTs). Soluble PFTs are recruited to the plasma membrane surface by binding to sugar, lipids or receptors molecules. The PFTs concentrate on the membrane surface, and then two pathways are alternatively taken to form the final membrane pore. In one pathway applicable to most β-PFTs, PFTs oligomerize on the membrane surface, and produce a pre-pore intermediate structure. In another pathway applicable to most α-PFTs, PFTs oligomerize and insert into the plasma membrane coordinately by a mechanism of sequential oligomerization, which forms a partial pore or complete pores, and both are active. In both pathways of α-PFT and β-PFT, formed transmembrane pores present distinct characteristics, and trigger cell responses.

Based on the main secondary structure of the transmembrane motif, PFTs can be categorized as α-PFTs that form α-helical transmembrane pore and β-PFTs that form β-barrel pores. The α-PFTs use amphipathic helices to construct pores in the target membrane, where β-PFTs use β-barrel to transverse the membrane. As described in Table 1, nine subfamilies of PFTs have been identified. Distinct structural characteristics of the PFT family lead to different insertion mechanisms (Figure 1). Generally, the inactive form of PFTs is secreted as a soluble monomer, which then converts from a soluble state to a transmembrane form (Figure 2). The understanding of the fine membrane pore-formation process is still not entirely clear and needs special techniques to track pore formation kinetics.

### 2.1. α-PFTs Family

The structures of many α-PFTs have been determined by X-ray crystallography and electron microscopy. Among five α-PFTs subfamilies, three subfamilies including colicin subfamily, cytolysin A subfamily and actinoporin subfamily, are the most studied PFTs; one subfamily of RTX family is α-PFTs with limited knowledge; the last subfamily is CAMP, which was recently discovered and presents a unique structure. For some α-PFTs, monomeric PFTs bind to the membrane in a step that precedes pore formation, and α-helices are usually used to punch pores and insert into the membrane.

#### 2.1.1. The Colicin Subfamily

Colicins are typical α-PFTs and are generally produced by and toxic to *Escherichia coli (E. coli)*. In *E. coli* strains, 25 different colicin members have been identified, among which colicins E1, A, B, N, Ia, Ib, K, 5, 10 are pore-forming colicins [62]. These PFTs are used to selectively eradicate other bacterial populations in the microbial community by punching holes in the inner membranes of these bacteria [63]. The first PFT structure described was colicin A, presenting a unique model for helices inside-out folded when inserted into lipid bilayers. The smallest pore-forming colicin is colicin N. These membrane pore-forming motifs of colicin consist of a bundle of α-helices. It was reported that during the pore formation, except colicin A, colicin E and Ia also results in an “umbrella” conformation with oligomerized dimers or higher-order assemblies, which was shown by electron paramagnetic resonance (EPR) spectra [64]. Moreover, combined with electrostatic binding to the surface and hydrophobic interaction, colicins finally form mature membrane pore. An earlier study suggested that some helices are buried within colicins and form a hydrophobic hairpin loop structure in soluble state. Upon spontaneously binding to the target membranes, the hydrophobic α-helical hairpins are exposed to form the transmembrane umbrella-like pores and subsequently insert into the lipid bilayer of the membrane [65]. Meanwhile, the insertion of the hydrophobic hairpin also could activate protein polymerization. The low pH and negatively charged micro-environment at the membrane interface may trigger this unfolding event, forming a nonspecific voltage-dependent pore supported by single-molecule study [66], leading to membrane depolarization and ultimately cell death. The exact structure and stoichiometry of colicin pore needs further exploration.

Besides the *E. coli* toxin, other bacterial toxins and eukaryotic proteins also adapt the colicin-fold structure. For example, the diphtheria toxin mediates the pore formation by colicin-fold domain to facilitate the translocation of the catalytic subunit of the toxin across the endosomal membrane, and endosomal acidification triggers conformational changes of toxin domain and ultimately insertion into the cytoplasm [67]. *Bacillus thuringiensis* produced pore-forming insecticidal Cry toxin also exhibits colicin-fold [68], a homologous ancestry fold with colicins to form bacterial type III secretion systems [69]. In the apoptotic pathway of eukaryotes, a phage-derived pore-forming BAX and BCL-2 homologous antagonist/killer (BAK) proteins accumulate on the mitochondrial and conduct colicin-like structural folds to mediate cytochrome c release, leading to cell death [70].

#### 2.1.2. The Cytolysin a Subfamily

The cytolysin A subfamily includes cytolysin A (ClyA, also known as HlyE, or SheA), non-hemolytic tripartite enterotoxin (Nhe), and the B component of hemolysin BL enterotoxin (Hbl), constituting another distinct class of α-PFTs [71]. Cytolysin A is produced by certain strains of *E. coli*, and homologs of ClyA are also produced by *Salmonella enterica* and *Shigella flexneri.* Another cytolysin A subfamily member, Nhe and Hbl, is produced by *Bacillus cereus*. ClyA monomer structure and pore complexes have been determined [15]. Accordingly, large and remarkable conformational changes involving 80% of residues and unique pore-forming mechanisms are observed. In solution, monomer ClyA displays an elongated almost entirely α-helical secondary structure, and a short hydrophobic β-tongue. Upon membrane interaction, the β-tongue detaches from the protein and approaches the cholesterol-rich membrane, triggering conformational changes and rearrangement of N-terminal amphipathic α-helices and leading to its insertion inside the membrane [15]. Moreover, twelve monomers oligomerize together to form a ring-like helical barrel pore [14,15] (Figure 1A). The detergent digitonin can induce trimer-like intermediate oligomer of ClyA, even without the formation of mature pore. However, whether trimers are formed in the regular pore formation process needs to be further confirmed [16].

The members of the Cytolysin A subfamily adopt similar pore-formation mechanism although with various numbers of protomers composition. Cytolysin A subfamily contains single, two, and three-component members. *E. coli* ClyA contains a single component, and YaxAB presents two-component. YaxAB from *Yesinia enterolitica* and XaxAB from *Xenorhabdus nematophila* have been determined to provide similar structure with ClyA but demonstrate low sequence identity [20,72]. In mature YaxAB pore, two-component proteins (bipartite PFTs) are arranged as ten symmetrical heterodimers showing a spoked rim from the top [19], while XaxAB possesses 12–15 heterodimers. XaxA stabilizes and activates XaxB, while XaxB is responsible for puncturing the membrane [20]. Except for single and bipartite PFTs, tripartite PFTs also have been solved; for example, Hbl is comprised of Hbl-L1, Hbl-L2, and Hbl-B proteins while Nhe is comprised of NheA, NheB, and NheC. However, the assembly mode of these tripartite ClyA subfamilies is unclear. Recently, another tripartite ClyA subfamily toxin, AhlABC, was identified in *Aeromonas hydrophila (A. hydrophila)*, and it showed all three components were involved in causing maximum cell lysis. AhlC tetramer first disassembles into monomers to bind membrane and recruits AhlB. Then AhlB undergoes a large conformational change from β-tongue to an extended α-helix and forms an active pore when binding AhlA [73]. Besides, there are differences in helix length among these PFT subfamily members. For example, the helix is much shorter in Nhe A, a component of the tripartite Nhe toxin [17], resulting in different of pore formation process. Formation of ClyA pore requires concentrated α-helices by a mechanism of circular oligomerization. Twelve monomers of ClyA oligomerize together and undergo large conformational changes to form a ring-like helical barrel pore [15].Another alternative and non-classical pore formation mechanism is that *E. coli* ClyA forms a soluble prepore within the outer membrane vesicles (OMVs) [74]. For *Vibrio cholerae* cytolysin (VCC), a hemolysin subfamily member also adopts a similar OMV-mediated delivery mechanism [74]. However, this OMV-mediated toxin secretion and delivery system have not been well illustrated yet.

#### 2.1.3. The Actinoporin Subfamily

Actinporins toxins are produced by sea anemones. These toxins represent another distinct subfamily of α-PFTs. Actinporins toxin protein of venom can paralyze predators and guard themselves by punching pores in the target cell membrane. The actinporin subfamily, including equinatoxin II (EqtII), produced by *Actinia equina*, fragacea toxin C (FraC) produced by *Actinia fragacea*, and sticholysin I and II (Stn I and Stn II) produced by *Stichodatyla helianthus* have been well studied. These proteins are composed of a core structure with β-sandwich and two flanking with α-helices. Upon binding to the lipid membrane by specific recognition of sphingomyelin (SM) or phase-separated lipid membranes, the N-terminal amphipathic helix detaches from the β-sandwich and inserts into the membrane lipid bilayer to form transmembrane function pore. Eventually, 3–4 monomers oligomerize on the membrane surface and simultaneously inserted into the membrane to form transmembrane pore with the membrane lipids rearrangement detected by differential scanning calorimetry and atomic force microscopy [75] but without a pre-pore intermediate state. Another atypical PFT with a similarβ-sandwich structure with actinoporin of α-PFTs is thermostable direct hemolysin (Tdh) produced by *Vibrio parahaemolyticus* [76,77]; Intra-protomer disulphide bond formation during Tdh folding/assembly process facilitate Tdh oligomerization. However, whether it is an α-PFTs still unclear.

The pores stoichiometry formed by these actinoporin subfamily varies from tetramers, such as Eqt II [78] and Stn II [12] to octamer such as FraC [79]. X-ray crystal structures of FraC in the form of monomeric, dimeric and octameric have been determined. It illustrated that the octameric FraC pore is assembled with sphingomyelin at a ratio of 1:1. Here, sphingomyelin acts as a receptor and a cofactor to complete the assembly of the mature pore. Besides, the N-terminal amphipathic helix of FraC undergoes a conformational change near the protomer state in pores. FraC pore comprises eight protomers (Figure 1A) with the hydrophobic motif towards oligomer, and hydrophilic motif towards the pore. This α-helical bundle structure suggested that actinoporin protomers might directly assemble into a pore conformation without a pre-pore intermediate state, although the mechanism of the actinoporin subfamily is not completely clear. In manyα-PFTs, it is coupled and synchronous events for oligomerization and membrane insertion to finally form transmembrane pore [80]. Hydrophobic residues located external lumen towards the membrane lipid, while hydrophilic residues located interior lumen towards water molecules.

#### 2.1.4. Other α-PFT Families

The structure and pore-formation mechanism of PFTs have been focused on and studied over several decades. Some unclassified PFTs orphans, such as the repeats-in-toxin (RTX) subfamily, represent a unique class of bacterial exoproteins possessing numerous glycine-rich repeat units (G–G–X–G–(N/D) –D–X–(L/I/V/W/Y/F)–X) at the C-terminus of each protein. This subfamily includesα-hemolysin (HlyA) from *E. coli*, Adenylate cyclase-hemolysin (CyaA) from *Bordetella pertussis* [81], and the multifunctional autoprocessing repeats-in-toxin (MARTX) from *A. hydrophila* and other pathogens [82].These RTX motifs exhibit intrinsically elongated disordered coil in Apo state, while exhibit rigid fold in the calcium-binding state, which is involved in the calcium-dependent secretion process for unidirectional export through the secretory channel [83]. This conformation change applies to bacterial species producing RTX. The calcium-binding RTX domain of the adenylate cyclase toxin (CyaA), produced by *Bordetella pertussis*, requires sub-millimolar calcium concentrations to active CyaA toxins translocation across the plasma membrane [84].

In the RTX subfamily, the number of repeats is different among their subfamily members. These RTXs form a parallel β-roll conformation present by a mean of a right-handed spiral [85,86]. Other factors, such as membrane lipid composition and concentration, affect RTX toxin perturbations to membranes. Therefore, pore-formation by RTX toxins may be a complex dynamic process involving membrane remodeling. Studies describing the pore-forming stoichiometry and mechanism by the RTX subfamily are limited and remained to be solved.

Christie, Atkins, and Munch-Petersen (CAMP) is another unique pore-forming toxin produced by *Streptococcus uberis*, *Propionibacterium acnes*, *Streptococcus agalactiae*, and *Mobiluncus curtisii*. Recently, the soluble form of CAMP structure from *Streptococcus agalactiae* and *Mobiluncus curtisii* was determined and they reveal a unique bacterial toxin [21,22]. This CAMP subfamily presentsα- helices bundled with N-terminal 5 and C-terminal 3-helix. However, its interaction with the membrane remains a challenge. CAMP may bind to the GPI anchored cell membrane’s glycosyl moieties to promote CAMP N-terminus insertion into the membrane. The mechanism of CAMP toxin interaction with the target membrane needs further explored. Deciphering this mechanism will facilitate a better understanding of pore-formation and its co-hemolytic activity by this subfamily of PFTs.

### 2.2. β-PFTs Family

β-PFTs are secreted by a wide variety of pathogenic bacteria, and are more extensively studied for several decades. Among β-PFTs, three are the most studied PFTs, including hemolysins, aerolysins, cholesterol-dependent cytolysins (CDCs), and one is relatively homologous membrane attack complex/perforin (MACPF) subfamily. β-strands are responsible for form β-barrel and insert into the membrane. The hydrophobic residues of the transmembrane domain are away from the pore core, and towards membrane lipids.

#### 2.2.1. The Hemolysin Subfamily

*S. aureus* PFTs contribute to pathogenesis in different ways by interacting with distinct surface proteins, particularly in the immune system leading to cell death and bacterial dissemination, including α-hemolysin (Hla) with a single component assembling into heptameric pores and γ-hemolysin AB (HlgAB), HlgCB, Panton-Valentine leukocidin (PVL), leukocidin ED (LukED), and leukocidin AB (LukAB or LukGH) with two components assembling into octameric pores with four copies of each subunit [87]. Besides, necrotic enteritis toxin B (NetB) and δ-toxins from *Clostridium perfringens* [32], cytolysin from *Vibrio cholera* [88], and hemolysin from *Vibrio vulnificus* [34] are also the prominent members of hemolysin subfamily in the β-PFT, which oligomerize into small β-barrel pores.

Most hemolysin subfamily members have been crystallized in the soluble form or in the pore conformation, presenting structural characteristics of β-PFT families. For example, *S. aureus* hemolysins present a rather compact structure in solution, and the pre-stem domain composing of a three-stranded β-sheet is fastened by hydrogen bond and located against the protein core. Upon oligomerization, the pre-stem was released and detached from the protein core to generates a 14-stranded β-barrel by anti-parallel β-hairpin and neighboring hairpins, leading to a ring-like heptameric pre-pore structure. This outer hydrophobic domain of β-barrel spontaneously inserts into the membrane in a concerted manner to form transmembrane pores. Another possibility is that membrane insertion and amphipathic β-barrel formation are simultaneous events. The crystal structure of the VHH lectin domain showed a heptameric ring arrangement similar to VCC [34].

#### 2.2.2. The Aerolysin Subfamily

A second subfamily of β-PFTs is the aerolysin subfamily (aβ-PFTs). The first known member of the aerolysin subfamily, aerolysin, is produced by Gram-negative *Aeromonas* spp., and related subfamily members present in bacteria, plants, and eukaryotes and throughout all kingdom of life. For example, ε-toxin and enterotoxin produced by *Clostridium perfringens,* α-toxin produced by *Clostridium septicum*, monalysin produced by *Pseudomonas entomophila*, parasporin produced by *B. thuringiensis*, enterolobin produced by *Enterolobium contortisiliquum*; lysenin from the earthworm, biomphalysin produced by the snail *Biomphalaria glabrata*; βγ-CAT from the frog *Bombina maxima* are also the members of the aerolysin subfamily. While bacterial aβ-PFTs are involved in killing host cell or punching roles in other species, eukaryotic members of aβ-PFTs play a role in defense against parasites or pathogens. In the aerolysin subfamily members, primary sequences are diverse; however, crystallographic studies have revealed remarkable structural similarities among aerolysins members, including ε-toxin, enterotoxin (CPE), α-toxin, hemolytic lectin (LSL), hydralysin toxins, monalysin, and the lysenin toxins. This aβ-PFTs subfamily was revealed and characterized to include a common structural fold consisting of two concentric β-barrels.

The aerolysin fold protein, abundant in β-structure, is multidomain although relatively small with molecular weights 30 and 60 kDa. Usually, aβ-PFTs are composed of one or more N-terminal receptor-binding domains (RBDs) and C-terminal the pore-forming module (PFM). However, RBDs located at the C-terminus are also found, such as in lysenin and enterotoxin structure. There is an exception that monalysin produced by *Pseudomonas entomophila* exhibits globular single domain structure and lack the RBD [47]. Monalysin pro-pore forms a stable doughnut-like 18-mer complex with two disks, in which the membrane-spanning region is fully buried. This conformation is different from other β-PFTs that receptor-dependent for membrane interaction [47]. The structural PFM, essential for pore formation, is a common conserved feature of aβ-PFTs while RBD shows sequence differences.

The aerolysin in solution presents highly elongated conformation. Aerolysin is initially synthesized as a protoxin with a C-terminal extension where it oligomerizes into a heptameric ring-like structure after cleavage of C-terminal peptide [39]. The intermediate pre-pore and mature pore structures of the aerolysin in the pore-forming process have been determined by combining X-ray crystallography and cryo-electron microscopy (Cryo-EM) [40]. In general, the monomers assemble into a pre-pore structure with heptameric oligomer docking on the membrane surface. Then the pre-stem loops refold into transmembrane amphipathic β-hairpins to form a β-barrel pore. Eventually, the pre-pore twists sideways to transit into the transmembrane pore by a swirling mechanism that is likely to be shared by other aerolysin subfamilies. During this period, it undergoes spectacular rearrangement of the aerolysin pre-pore from an inverted mushroom shape to a disk-like extracellular structure with a central β-barrel stem in the view of a top to the bottom [3]. Aerolysin pores composed of six to nine protomers are relatively small, with the diameter ranging from 1–4 nm. A similar pore architecture was also observed in the hemolytic lectin CEL-III from *Cucumaria echinata* [89]. A recent study revealed another novel protein fold and pore-formation mechanism by aerolysin. The oligomerized monomer can form two highly stable concentric β-barrels with zipper-like. This aerolysin forms a final pore in a lipid bilayer by a mechanism of piston-like puncturing [39]. The pore-forming mode of aerolysin is shared with many other β-PFTs. But it still needs further investigation to confirm their interaction mechanism.

In membrane pore formation, amphipathic β-barrels are stabilized and they are fixed in position by a distinct mechanism. Charged residues of the transmembrane β-hairpin in *S. aureus* Hla and hydrophobic residues of transmembrane β-hairpins in aerolysin are responsible for anchoring the barrel preventing movement. The latter adopted a rivet-like configuration in the bilayer core. Furthermore, the β-barrels lumen of aerolysin is also full of charged residues, which is different from those in *S. aureus* Hla. Besides, *Clostridium perfringens* epsilon toxin (Etx, ε-toxin) is also a member of aβ-PFTs. The overall structure of the Etx pore resembles aerolysin with the β-barrel spanning the height of the pore (Figure 1B). ε-toxin pore suggests conserved and concentric double β-barrel with heptameric oligomerization. It is also devoid of a vestibular region observed in the α-hemolysin subfamily, and the protomer consists of β-hairpin, cap, and RBD domain [36]. During pore formation, Etx monomer undergoes large conformational changes, among which β-hairpin with insertion loop unfolds to create the inner β-barrel while cap domain moves away from inner β-barrel.

#### 2.2.3. The Cholesterol-Dependent Cytolysin Subfamily

The cholesterol-dependent cytolysin (CDC) subfamily is a large subfamily of β-PFTs, which form large oligomeric pore complexes responsible for disrupting cellular membranes [90,91]. This subfamily mostly produced by the Gram-positive bacteria, including perfringolysin O (PFO) of *Clostridium perfringens* [92], suilysin (SLY) of *Streptococcus suis*, streptolysin O (SLO) of *Streptococcus pyogene,* intermedilysin (ILY) of *Streptococcus intermedius*, listeriolysin O (LLO) of *Listeria monocytogenes* [53], anthrolysin O (ALO) of *Bacillus anthracis* [93], and pneumolysin (PLY) of *Streptococcus pneumoniae*.

The structures of CDCs have been determined by X-ray crystal, electron microscopy and AFM analysis. Different from hemolysin and aerolysin-like small β-PFT contributing single β-hairpin to form β-barrel pores, large pore-forming β-PFT, each CDC protomer contributes two amphipathic β-hairpins to transmembrane β-barrel. CDCs assembled into very large ring-like structures composing of around 30 to 50 protomers. However, the factors and mechanisms triggering the transmembrane pores are still not entirely clear. A conserved F/Y-F/Y-Xn-YGR motif with the CDCs is reported critical as the sensor to initiate the prepore-to-pore transition [94].

All CDCs have the same domain structure as shown by sequence comparison, and thus inserting into target membranes could be by the same way. PFO is an elongated β-sheet-rich multidomain protein, and the relatively stable interface between the D3 and D1, 2 play a key role in pore formation by PFO and even entire CDCs [95]. All CDCs are secreted, except pneumolysin, which is released after bacterial autolysis or antibiotic therapy. Pneumolysin (PLY) is another CDC, with typical characteristics. The structure of soluble monomer and ring-like PLY pore have been determined by X-ray diffraction [96] and cryo-EM, respectively, which illustrate the mechanism of membrane insertion and mature pore formation by time-lapse AFM [57]. Monomer PLY assemble into rings on the membrane surface side by side, then D3 helices refold and trigger the refold of the neighbor monomer. The unfolding β-hairpins traverse the hydrophobic membrane and merge into one large 168-strand β-barrel, which is irreversible.

In general, the monomer CDC presents a central β-sandwich flanking with two pairs of short α-helices. Upon oligomerization, α-helices undergo a drastic prion-like conformational transition from α-helix-toβ-strand.80–200 β-strands insert into the membrane to form a giant β-barrel. This mode of action is also observed in hemolytic lectin CEL-III of the sea cucumber [89]. By an oligomerization mechanism of sequential addition, CDCs produce not only absolute ring pores but also produce arc-like structures in the state of pre-pore, and both states are active, which was also confirmed for SLY [97]. Moreover, β-strands alignment is essential for membrane pore assembly and formation. In the pre-pore complex, β-hairpins are highly dynamic, while in the pore state, it exhibits a locked β-barrel, and β-strands produce a 20° tilt to the membrane [98]. One study by AFM revealed that CDCs undergo conformational elongation on the membrane surface during the pre-pore-pore transition [99]. The CDC pre-pore transits to pore by undergoing a collapse. This process requires the tilting of β-strands to the membrane and the rotation of the toxin core domain by the swirling mechanism, which is also reported in aerolysin collapse [40]. A similar architecture was also observed for mammalian perforins and membrane attack complex components [91], suggesting these proteins may be ancient relatives. The understanding of the pore-forming mechanism of CDCs can also contribute to other relative proteins. Besides, during the process of pore-formation, membrane insertion may be accompanied by membrane lipid organization. More studies are warranted to reveal the mechanism for host-PFTs interactions at molecular levels.

## 3. Diversification of Interaction of PFTs with Membrane Components

PFTs produced by different organisms target distinct host membrane by interacting with membrane sugar, lipids, and protein receptors or receptor-like molecules by recognizing specific structural motifs (Figure 2). Efficient interaction of PFT with cell membrane receptors is a critical initial step to drive PFTs self-assembly, the membrane pore formation and a subsequent array of signaling cascades and cellular responses [2]. In this process, PFTs first bind to the membrane to concentrate monomer PFTs on the membrane surface, facilitating the self-assembly and membrane insertion action of the PFT protomers.

Bacterial PFTs or PFPs that target bacteria often bind to glycans that covalently coupled membrane associated proteins or in the glycosyl phosphatidyl inositol (GPI) region. For example, VCC of hemolysin subfamily was shown to bind cell surface glycosylated proteins through β-trefoil and β-prism domain, among which β-prism can augment the binding of VCC to the cell membrane by surface glycans to facilitate further pore oligomerization and formation [100,101]. Another study indicated a sialoglycoprotein, glycophorin Bis a receptor for VCC [102]. VVH recognizes various cell surfaces by binding to different sugars such as glycerol, N-acetyl-D-galactosamine (GalNAc), and N-acetyl-D-lactosamine (LacNAc) [34], suggesting a versatile mode of recognition for VVH invading host cell. Aerolysin of aerolysin subfamily binds to the N-linked glycan and the GPI anchor on cell the membrane by its N-terminal protruding domain of the protein core [103,104]. Besides, ε-toxin of aerolysin subfamily binds to hepatitis A virus cellular receptor 1 (HAVCR1), an O-linked glycoprotein [105]. In another receptor, the tetraspan membrane proteolipid myelin and lymphocyte protein (MAL), the second extracellular loopis critical for binding and cytotoxicity of ε-toxin [36]. Intermedilysin (ILY) of CDC subfamily binds to CD 59 on the human cells, which is a GPI-anchored protein [106]. CAMP subfamily toxins may also function through attaching to the GPI moiety on eukaryotic cell surfaces [21,107]. Colicin N of colicins subfamily first concentrates on the bacterial outer membrane by binding to lipopolysaccharides (LPS), then translocates to the inner membrane through porin proteins [108]. Besides, eukaryotic C-type lectins active immune response by binding to peptidoglycan carbohydrate of the gram-positive bacteria cell wall and oligomerize to form a hexameric membrane pore [109].

For membrane pore formation, some PFTs also prefer binding to lipids in the membrane and lipid type play an essential role in pore formation. In aerolysin subfamily, aerolysin has been revealed to bind to lipid rafts and lipid-anchored protein [110]. Lysenin specifically binds to sphingomyelin [111] abundant in lipid rafts. As a constitutive cofactor, sphingomyelin is involved in assembling the FraC pore of the sea anemone toxin [79]. The lipid-binding sites and lipid environments in this toxin modulate the affinity and specificity of membrane binding. In addition to glycosylated proteins and receptor binding, specific membrane lipids, including cholesterol and sphingolipid, can also regulate VCC’s pore-forming activity [112,113], however, it did not show significant structural specificity in the toxin-cholesterol interaction. *Vibrio cholerae* cytolysin (VCC) binds nonspecifically to membrane lipid bilayer via amphipathicity-driven partitioning and bind specifically to membrane phospholipid head group via VCC motif [113].

Cholesterol, another lipid abundant in lipid rafts, mediates CDCs oligomerization in lipid raft-like domains. Cholesterol is required for CDCs cytolytic activity. In CDC subfamily, PFO is extensively used to study the interaction of CDCs with membranes. The PFO oligomerization and pore formation depends on the host membrane’s cholesterol concentration. The composition and arrangement of membrane lipid is important to CDC pore formation [114]. It was reported that the lipid environment is critical for the interaction of LLO with cholesterol through 19F-NMR spectroscopy [115]. Such that pore formation regulation is achievable by changing the lipid composition. In specific membrane environments, the structure of L3 and possibly L2 of CDCs can facilitate the optimal binding of different CDCs [116]. The conserved undecapeptide ECTGLAWEWWR and threonine-leucine pair among CDC provides the key motifs for membrane binding [117] and PFO pore formation [118]. In addition to lipid rafts, lipids themselves can directly regulate membrane pore formation. For example, *E. coli* colicins of β-PFT showed an anionic lipid, cardiolipin, in the bacterial inner membrane [64], promoting the umbrella pore conformation.

In addition to sugars and lipids, some specific protein receptors of PFTs have been identified. For example, a disintegrin and metalloprotease 10 (ADAM10) could be a protein receptor that has high affinity for *S. aureus* α-hemolysin (Hla) and enables cytotoxic activity target to epithelial cells [119]. Cry, a biological insecticide produced by *Bacillus thuringiensis* binds on its receptor aminopeptidase N and cadherin-like proteins. Besides, for the host immune system, PFTs targets distinct immune cells by binding specific receptors. PVL targets neutrophils, even monocytes, and macrophages by binding to the C5a receptor (C5aRs) [120]. CD31 or PECAM-1 on endothelial cells is the specific membrane receptor for *Clostridium perfringens* β-toxin and essential for interaction [121]. In most cases, cholesterol on the target membranes has been shown as the receptor for the CDCs. Specific structural motifs of CDCs are responsible for recognizing and binding to cholesterol receptors. In other cases, intermedilysin (ILY) of CDCs, bind to CD59 receptor (a GPI-anchored protein) [106], in which cholesterol is not the direct receptor but still required to form membrane pore. The ILY crystals have been recently determined in soluble monomer and complex with human CD59 receptor, which defines two distinct interfaces. It also revealed that ILY-derived peptide inhibits pore formation through interfering binding between ligand and receptor [50,51]. Vaginolysin (VLY), a CDC produced by *Gardnerella vaginalis*, can bind to both CD59 and cholesterol as receptors [122]. In fact, CDCs bind glycan and cholesterol independently.

These characterizations of PFTs provide insight into that structure-guided design of PFTs-binding peptides and disruption of interaction with their target membrane components, offering a promise for therapeutic development.

## 4. Anti-Infection Therapeutic Strategies and Application Targeting PFTs

PFTs of most pathogenic bacteria’s ability to invade host makes it an attractive target for developing potent drugs that can withstand the acquired resistance observed in the conventional antimicrobial therapy.

The growing illumination of the structure and function of PFTs helps to develop antimicrobial drugs by targeting these proteins or their interaction with membrane receptors. Based on conformational rearrangements in PFTs during pore formation, it offers ways of screening small compounds; for example, Oroxylin A can inhibit the hemolytic activity of Hla by hindering the transmembrane pore assembly [123]. The soluble n-tetradecylphosphocholine (C14PC) compound can protect human immune cells against lysis by PVL and α-toxin [25]. Theoretically, these compounds can be used in the treatment of multidrug-resistant *S. aureus* infections. Furthermore, DNA aptamers, as a novel strategy, can also target *S. aureus* α-toxin [124]. Quercetin as a promising therapeutic candidate alleviated cytotoxicity by targeting SLY and subsequent inflammation for *Streptococcus suis* infection [125].

Given the specific binding of PFTs to receptors, synthetic GPI molecules and GPI analogs can inhibit pore assembly [126]. Also, by a similar mechanism, therapeutic antibodies have potential roles in hindering pore formation. It was reported in both nondiabetic and diabetic mice model, neutralization of α-toxin with anti-α-toxin monoclonal antibody had a therapeutic effect on *S. aureus*-infected injury [127]. Targeting of PFTs provides a novel therapeutic approach for bacterial infections. Similarly, the receptor could also be targeted. For example, CCR5 antagonist, maraviroc, blocks LukED-dependent cell death and confers resistance to *S. aureus* infection in CCR5-deficient mice model [128]. P2XR-receptor antagonist also prevented Hla-induced lysis by interfering with its interaction with membranes [129]. However, this approach targeting receptors as a therapeutic strategy is impractical as it may harm the immune system.

Developing recombinant toxoid vaccines by targeting PFTs is another strategy to reduce the toxicity of PFT and trigger immune responses. Based on toxin and pore structure, it is feasible to design attenuated vaccine by mutation, such as variants of NetB [130] and ε-toxin [131]. Recombinant leukocidin domain of *Vibrio vulnificus* hemolysin A was an effective toxoid to protect against *Vibrio vulnificus* in a mouse model [132]. In addition, *S. pneumoniae* Ply of the CDC subfamily has been targeted to develop promising pneumolysoid candidates, including Δ6PLY mutant [133] and PsaA [134] and CbpA [135]. Recent two PLY mutant PLY_D168A_ and PLY_Δ146/147_ by interfering with monomer refold and membrane insertion were reported no hemolytic activity, the later also can not bind to the membrane. These amino acid sites play a important role in ionic interaction between β-strands to stabilize the pore complex [57]. Nanotoxoid vaccines combining the non-toxic PFTs with antigen presentation, may have potential value infighting against antibiotic-resistant infections. For example, *S. auerus* Hla pores nanotoxoid triggers an effective immune response in vivo [136]. Biomimetic nanoparticles inhibit the cytotoxic effects of GBS β-hemolysin/cytolysin [137].

The Cry34Abl/Cry35Abl Cry toxin from *Bacillus thuringiensis* have been introduced to corn hybrids to provide protection from the western corm rootworm feeding via a pore forming mechanism [138]. Finally, the effect of pore formation on cell death can be applied as an interesting suicide gene therapy in tumor cells by transfecting the PFT into cancer cell lines. For example, moxetumomabpasudotox-tdfk (LUMOXITI^®^, AstraZeneca Pharmaceuticals LP), animmunotoxin chimeric composed of a pore-forming domain of Pseudomonas exotoxin A and an antibody that is responsible for targeting cell, has been approved for application in B-cell cancer by the FDA in 2018 [139].

## 5. Conclusions

PFT is a widespread virulence factor of pathogenic bacteria. Final results of pore formation modulate or kill host cells, leading to bacterial dissemination and growth. Extensive studies provide a general model of actions of diverse PFTs. Inactive, soluble PFTs undergo a conformation change to form mature and sophisticated transmembrane pores on the target cell membrane, although each of the PFT families has distinct structural folding and pore formation mechanism. Different members within specific PFT subfamily (α-PFTs and β-PFTs) have distinct structural features. Therefore, PFTs from distinct subfamilies sometimes mediate similar functional consequences. Furthermore, identifying PFTs receptor in membrane lipids, sugars, and proteins reveal specific interaction between host and pathogens. In summary, elucidating these structure mechanisms of membrane pore formation enables the designing of therapeutics including antibodies, drugs, peptides or nanotoxoids, and subsequent infection, particularly for multi-drug resistant strains.

The structural characterization of monomer PFTs and their identified membrane pore has increased the understanding of PFTs to some extent. However, more studies are required to explore further the membrane pore complexes and kinetic processes of pore formation to further reveal the implication of the PFTs for host-pathogen interaction.

## Figures and Tables

**Table 1:** Classification and details of pore-formation specific for each PFT family members.

PFT	Family	Receptor	PDB	Organisms	References
**α-PFT**					
Colicin A	Colicins	IM/OM	1COL	*E. coli*	[6]
Colicin N	Colicins	IM/LPS-OM	1A87	*E. coli*	[7]
Colicin Ia	Colicins	IM/OM	1CII	*E. coli*	[8]
Colicin E1	Colicins	IM/OM	2I88	*E. coli*	[9]
Colicin B	Colicins	IM/OM	1RH1	*E. coli*	[10]
EquinatoxinII (EqtII)	Actinoporins	Sphingomyelin	1IAZ	*A. equina*	[11]
SticholysinII (StnII)	Actinoporins	Sphingomyelin	1GWY	*S. helianthus*	[12]
Fragaceatoxin C (FraC)	Actinoporins	Sphingomyelin	4TSL, 4TSY	*A. fragacea*	[13]
Hemolysin E (HlyE)	CytolysinA	Cholesterol	1QOY, 2WCD, 6MRT	*E. coli*	[14,15,16]
Non-hemolytic tripartite enterotoxin (Nhe)	Cytolysin A	Cholesterol	4K1P	*B. cereus*	[17]
HaemolysinBL (Hbl)	Cytolysin A	Cholesterol	2NRJ	*B. cereus*	[18]
YaxAB	Cytolysin A		6EK4, 6EK7, 6EK8, 6EL1	*Y. enterolitica*	[19]
XaxAB	Cytolysin A		6GY6	*X. nematophila*	[20]
CAMP	CAMP	GPI-anchored proteins	5H6I 6JLC	*S. agalactiae* *M. curtisii*	[21,22]
α-hemolysin (HlyA)	RTX	β2 integrin		*E. coli*	
Adenylate cyclase-hemolysin toxin (CyaA)	RTX	-	2COL SASDCK9 SASDCL9	*B. pertussis*	[23,24]
MARTX	RTX	-	-	*A. hydrophila*	
**β-PFT**					
α-haemolysin (Hla)	Hemolysin	PC/ADAM10/ disintegrin	3M2L, 3M4D, 7AHL, 4YHD, 6U49, 6U4P, 6U3T	*S. aureus*	[25,26,27,28]
γ-hemolysin (Hlg)	Hemolysin	PC	4P1Y	*S. aureus*	[29]
Leukocidin (HlgACB, LukED)	Hemolysin	CCR5, CXCR1, CXCR2, CCR2,C5aR, DARC	3ROH, 4Q7G	*S. aureus*	[30]
Necrotic enteritis toxin B (NetB)	Hemolysin	Cholesterol	4H56	*C. perfringens*	[31]
δ toxin	Hemolysin	Monosialicganglioside 2 (G_M2_)	2YGT	*C. perfringens*	[32]
*Vibrio cholerae* cytolysin (VCC)	Hemolysin	Glycoconjugates	1XEZ	*V. choleraes*	[33]
*Vibrio vulnificus* hemolysin (VVH)	Hemolysin	gangliosides, N-acetyl-D-galactosamine, N-acetyl-D-lactosamine	4OWJ, 4OWK, 4OWL	*V. vulnificus*	[34]
α-toxin	Aerolysin	GPI-anchored proteins	1KHO	*C. perfringens*	[35]
ε-toxin (Etx)	Aerolysin	HAVCR1, MAL	1UYJ, 6RB9, 3ZJX	*C. perfringens*	[36,37,38]
Aerolysin	Aerolysin	GPI-anchored proteins (CD52)	1PRE, 3C0M, 3C0N, 3C0O, 5JZT	*Aeromonas* spp.	[39,40,41]
Hydralysin (Hln)	Aerolysin	-	-	*Cnidaria* spp.	
Enterotoxin (CPE)	Aerolysin	Claudin	3ZIW	*C. perfringens*	[42]
Lysenin	Aerolysin	Sphingomyelin	3ZXD, 3ZXG, 3ZX7, 5EC5, 5GAQ	*E. fetida*	[43,44,45]
Hemolytic lectin (LSL)	Aerolysin	Carbohydrates	2Y9F	*L. sulphureus*	[46]
Monalysin	Aerolysin		4MKO, 4MJT	*P. entomophila*	[47]
Perfringolysin O (PFO)	CDCs	Cholesterol	1PFO	*C. perfringens*	[48]
Suilysin (SLY)	CDCs	Cholesterol	3HVN	*S. suis*	[49]
Intermedilysin (ILY)	CDCs	Cholesterol, CD59	1S3R, 4BIK, 5IMW, 5IMT	*S. intermedius*	[50,51,52]
Listeriolysin (LLO)	CDCs	Cholesterol	4CDB	*L. monocytoge-nes*	[53]
Lectinolysin (LLY)	CDCs	Cholesterol, CD59	3LEI	*S. mitis*	[54]
Anthrolysin O (ALO)	CDCs	Cholesterol	3CQF	*B. anthracis*	[55]
Streptolysin O (SLO)	CDCs	Cholesterol	4HSC	*S. pyogenes*	[56]
Pneumolysin (PLY)	CDCs	Cholesterol	4ZGH, 5AOF, 5LY6, 5CR6, 5AOE	*S. pneumoniae*	[57,58,59]
Vaginolysin (VLY)	CDCs	Cholesterol	5IMY, 5IMT, 5IMW	*G. vaginalis*	[50]
Plu-MACPE	MACPF	-	2QP2	*P. luminescens*	[60]
Bth-MACPE	MACPF	-	3KK7	*B. thetaiotaomicron*	[61]

PFT, pore-forming toxin; IM, bacterial inner membrane; OM, bacterial outer membrane; LPS, lipopolysaccharide; *E. coli*, *Escherichia coli*; *A. equina*, *Actinia equina*; *S. helianthus*, *Stichodactyla helianthus*; *A. fragacea*, *Actinia fragacea*; *B. cereus*, *Bacillus cereus*; *Y. enterolitica*, *Yesinia enterolitica*; *X. nematophila*, *Xenorhabdus nematophila*; CAMP, Christie, Atkins, and Munch-Petersen; GPI, glycosyl phosphatidyl inositol; *S. agalactiae*, *Streptococcus agalactiae*; *M. curtisii*, *Mobiluncus curtisii*; RTX, repeats-in-toxin; *B. pertussis*, *Bordetella pertussis*; MARTX, multifunctional autoprocessing repeats-in-toxin; *A. hydrophila*, *Aeromonashy drophila*; PC, phosphatidylcholine; ADAM10, disintegrin and metalloproteinase domain-containing protein 10; *S. aureus*, *Staphylococcus aureus*; CCR5, CC-chemokine receptor type 5; CXCR1, CXC-chemokine receptor type 1; C5aR, C5a receptor; DARC, Duffy antigen receptor for chemokines; *C. perfringens*, *Clostridium perfringens*; *V. cholerae*, *Vibrio cholerae*; *V. vulnificus*, *Vibrio vulnificus*; HAVCR1, hepatitis A virus cellular receptor 1; *E. fetida*, *Eisenia fetida*; *L. sulphureus*, *Laetiporus sulphureus*; *P. entomophila*, *Pseudomonas entomophila*; CDC, cholesterol-dependent cytolysin; *S. suis*, *Streptococcus suis*; *S. intermedius*, *Streptococcus intermedius*; *L. monocytogenes*, *Listeria monocytogenes*; *S. mitis*, *Streptococcus mitis*; *B. anthracis*, *Bacillus anthracis*; *S. pyogenes*, *Streptococcus pyogenes*; *S. pneumoniae*, *Streptococcus pneumonia*; *G. vaginalis*, *Gardnerella vaginalis*; MACPF, membrane attack complex component/perforin; *P. luminescensis*, *Photorhabdus luminescens*; *B. thetaiotaomicron*, *Bacteroides thetaiotaomicron*; PDB for which some structural data are available for the monomer and/or pore state; the list of receptors for each toxin is not exhaustive; “-” means unknown.

## Data Availability

Not applicable.
